# Staging laparoscopy and peritoneal cytology in patients with early stage gastric adenocarcinoma

**DOI:** 10.1186/s12957-020-01813-y

**Published:** 2020-02-17

**Authors:** Casey J. Allen, Alisa N. Blumenthaler, Prajnan Das, Bruce D. Minsky, Mariela Blum, Sinchita Roy-Chowdhuri, Jaffer A. Ajani, Naruhiko Ikoma, Paul F. Mansfield, Brian D. Badgwell

**Affiliations:** 1 Department of Surgical Oncology, The University of Texas MD Anderson Cancer Center, 1400 Pressler Street, FCT17.6010, Houston, TX 77030 USA; 2 Department of Radiation Oncology, The University of Texas MD Anderson Cancer Center, Houston, TX USA; 3 Department of Gastrointestinal Medical Oncology, The University of Texas MD Anderson Cancer Center, Houston, TX USA; 4 Department of Pathology, The University of Texas MD Anderson Cancer Center, Houston, TX USA

**Keywords:** Stomach, Cancer, Carcinomatosis, Peritoneal staging, Survival

## Abstract

**Background:**

Staging laparoscopy and peritoneal cytology can detect occult metastatic disease prior to treatment of gastric cancer. The yield of peritoneal staging in patients with early stage disease is lacking. We assess the yield of peritoneal staging in early stage gastric cancer and its impact on survival.

**Methods:**

Data were obtained from a prospective database of patients who underwent staging laparoscopy and peritoneal cytology for gastric cancer at our institution between July 1995 and July 2018. Clinical stage was determined by endoscopic ultrasound, and early stage was defined as cT1-2 and cN0. Rates of positive cytology and carcinomatosis at time of laparoscopy were obtained. Univariate analyses were used to compare groups, and Kaplan-Meier survival analyses were used to assess survival outcomes.

**Results:**

Eight hundred sixty-seven patients underwent staging laparoscopy and peritoneal cytology; 56 were defined as early stage. Age was 61 ± 12 years, 66.4% were male, and 62.3% were white. Of the patients with early stage disease, 17.9% had either gross carcinomatosis (10.7%) and/or positive peritoneal cytology (10.9%). All cases of peritoneal disease were in patients with cT2 disease. There were no differences in age, gender, or race based on peritoneal disease (all *p* > 0.05). The presence of carcinomatosis or positive cytology significantly affected overall survival (*p* < 0.001), regardless of clinical T or N stage.

**Conclusions:**

Peritoneal staging identifies metastatic disease in a significant number of patients with early stage disease. Given its poor prognosis and alternate therapy options, independent staging laparoscopy and peritoneal cytology should be considered in patients with early stage gastric adenocarcinoma.

## Introduction

Gastric adenocarcinoma (GA) remains one of the most common causes of cancer and cancer-related death worldwide [1, 2]. It is estimated that in 2018, over 26,000 new cases of gastric cancer will be diagnosed in the USA [1]. The peritoneum represents one of the most common sites of metastatic disease and recurrence in GA patients, and the presence of peritoneal disease carries a dismal prognosis [3–6]. The reported median survival for patients with peritoneal disease ranges between 6 and 15 months [4, 7–10]. Multiple studies have demonstrated the poor survival rates in patients with positive peritoneal cytology (PC), with or without macroscopic disease, at the time of definitive resection [4, 11–13]. The survival rates for patients with peritoneal disease on staging laparoscopy (SL) or positive PC is similar to patients with other metastatic disease identified on preoperative imaging [12]. As patients with peritoneal disease are unlikely to benefit from definitive resection, the National Comprehensive Cancer Network (NCCN) guidelines currently recommend systemic chemotherapy or best supportive care for those patients with known peritoneal disease [14].

There are multiple theories as to how peritoneal spread occurs in gastrointestinal cancers. It is considered to be a multi-step process, the first step of which involves free cancer cells becoming detached from the primary tumor. The most common mechanism of detachment is exfoliation of tumor cells from a primary tumor that has invaded the serosa [15]. Thus, it seems logical that SL and PC would be most valuable in only locally advanced tumors. Current NCCN guidelines recommend SL and PC in any T1b or higher tumors. However, the yield of SL and PC in patients with early stage disease (cT1-cT2, cN0) is unclear. The goal of our study was to evaluate the incidence of +SL/PC in patients with early stage disease, as well as its impact on survival.

## Methods

The Institutional Review Board of the University of Texas MD Anderson Cancer Center approved the study. A prospectively maintained database of gastric and gastroesophageal cancer patients within the Department of Surgical Oncology was queried. We analyzed data from patients with gastric cancer, who underwent SL and PC at our institution from July 1, 1995 to July 1, 2018***.*** Patient selection and variables collected were similar to those in a previous study by our group [5, 16, 17]. Only patients with pathologically diagnosed adenocarcinoma of the stomach who underwent SL and PC were included. The patient and tumor characteristics collected were age, sex, race/ethnicity, histological grade, the presence of signet ring cells, linitis plastica, gross peritoneal metastasis on SL, and positive PC. Clinical T and N status were determined mainly via endoscopic ultrasound (EUS) performed by experienced endoscopists at our facility. Early stage disease was defined as cT1-cT2 and cN0.

Patients were treated according to our institution’s treatment algorithm based on their disease status. Patients considered to be potentially resectable (negative SL/PC) underwent induction chemotherapy, followed by chemoradiation, restaging, and then attempted resection. Preoperative chemotherapy and chemoradiation techniques at our institution have been previously described [5, 16, 17]. Following resection, our standard surveillance practice is to perform 4–6 month follow-up with imaging. For those that were found to have peritoneal metastasis on initial SL/PC, systemic chemotherapy is the standard approach to treatment. These patients were treated prior to the standardization of our HIPEC program and thus did not receive intraperitoneal chemotherapy.

### Laparoscopy staging, peritoneal lavage, and cytological sampling

**Fig. 1:**
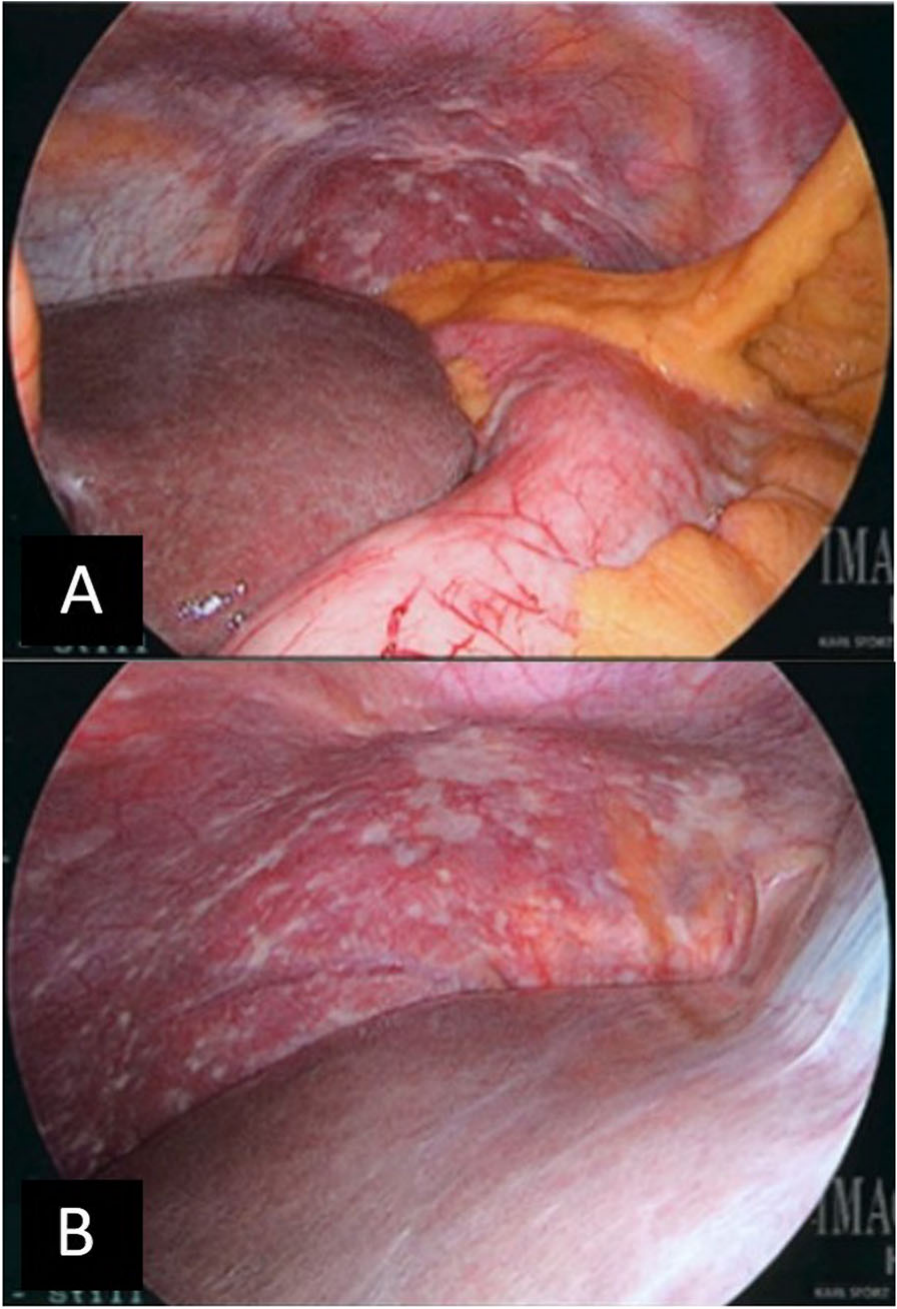
Gross carcinomatosis on staging laparoscopy. **a** Patient with mid-gastric adenocarcinoma with serosal invasion and peritoneal carcinomatosis. **b** Patient with distal gastric adenocarcinoma and gross peritoneal carcinomatosis identified on staging laparoscopy

Independent SL and PC are a part of the standard staging algorithm for any patient with a clinical stage T2 lesion or greater. At times, patients with T1 lesions will undergo SL and PC if there are high-risk features, or at the time of laparotomy and attempted resection. SL and PC were performed prior to any definitive treatment with the standard technique used at our institution, as previously described [5, 6, 16–20]. The peritoneal cavity was inspected for gross evidence of metastasis. Any macroscopic peritoneal lesions were biopsied and sent for permanent pathological examination. Figure 1 provides an example of gross peritoneal spread on SL. Macroscopically positive laparoscopy was defined as a positive biopsy of a visualized peritoneal lesion. After gross inspection of the peritoneal cavity, peritoneal cytological sampling was performed. PC was considered positive in any case of “malignant cells” or “adenocarcinoma” on hematoxylin and eosin (H&E) staining, and/or “atypical cells” or “suspicious for malignancy” on H&E with confirmatory immunohistochemistry (IHC) staining [21, 22]. Although a wide panel of immunostains including both epithelial and mesothelial cell markers are available, MOC-31 reliably differentiates adenocarcinoma [23]. Pathologists at our institution have been using IHC in our cytology specimens since the early 1990s and our practice has been relatively stable in this time period [24–27].

### Statistical analysis

Data are reported as mean ± standard deviation if normally distributed or median (95% confidence interval) if not. Differences were compared with Student’s *T* test for parametric data and Mann-Whitney *U* test for nonparametric data. Categorical data were compared with Pearson’s chi-square; if cell counts were < 5, Fisher’s exact test was used. Kaplan-Meier survival analyses were used to evaluate the implication of +SL/PC on overall survival (OS). *P* values < 0.05 were considered statistically significant. Statistical analyses were performed using SPSS version 24 (IBM Corporation; Armonk, NY).

## Results

A total of 867 patients underwent SL and PC and met study inclusion criteria. Age of the cohort was 61 ±12 years, 66.4% were male, and 62.3% were white. The majority of patients were cT3 (79.7%), cN1 (51.4%), or of poorly differentiated histological grade (77.7%). Fifty-six patients presented with early stage GA (cT1-cT2, cN0).

Table 1 displays the demographic and clinicopathologic characteristics of the cohort, comparing early stage GA patients to late stage GA patients. Late stage patients were more likely to be male (68.1% vs 42.9%, *p* < 0.001) and white (63.7% vs. 41.1%, *p* = 0.004). Of all patients with early stage who underwent peritoneal staging, 10 (17.9%) were found to be either grossly positive for carcinomatosis (10.7%) and/or have positive PC (10.9%).

**Table 1:** Demographic and clinicopathologic characteristics of patients with early and advanced GA (*n* = 867)

		T1-2, N0 (*n* = 56)	T3-4, N+ (*n* = 811)	*p* value
Age, years		63 ± 13	61 ± 12	0.267
Gender	Male	24 (42.9%)	552 (68.1%)	< 0.001
Race	White	23 (41.1%)	517 (63.7%)	0.004
	Hispanic	14 (25%)	152 (18.7%)	
	Black	5 (8.9%)	55 (6.0%)	
	Asian	9 (16.1%)	40 (4.9%)	
	Other/unknown	5 (8.9%)	47 (5.8%)	
Histology	Well differentiated	0 (0.0%)	3 (0.4%)	0.936
	Moderately differentiated	12 (21.4%)	177 (21.8%)	
	Poorly differentiated	44 (78.6%)	630 (77.7%)	
	Undifferentiated/anaplastic	0 (0.0%)	1 (0.1%)	
Signet ring cells		33 (58.9%)	379 (46.7%)	0.096
Linitis plastica		13 (25.0%)	93 (11.3%)	0.016
EUS T stage	T1a	0 (0.0%)	3 (0.4%)	< 0.001
	T1b	2 (3.6%)	0 (0.0%)	
	T2	54 (96.4%)	29 (3.6%)	
	T3	0 (0.0%)	691 (85.2%)	
	T4	0 (0.0%)	49 (6.0%)	
	T4a	0 (0.0%)	35 (4.3%)	
	T4b	0 (0.0%)	3 (0.4%)	
	Tx	0 (0.0%)	1 (0.1%)	
EUS N stage	N0	56 (100.0%)	273 (33.7%)	< 0.001
	N1	0 (0.0%)	446 (55.0%)	
	N2	0 (0.0%)	72 (8.9%)	
	N3	0 (0.0%)	20 (2.5%)	
Positive carcinomatosis		6 (10.7%)	169 (20.8%)	0.084
Positive cytology		6 (10.9%)	158 (21.9%)	0.059
LS or PC positive		10 (17.9%)	249 (30.7%)	0.049

Table 2 compares the tumor characteristics of those found with +SL/PC vs −SL/PC in patients with early stage GA (*n* = 56). All 10 cases of +SL/PC in early stage cancers were cT2 poorly differentiated tumors. We have reviewed the radiological features of the ten early clinical stage patients with +SL/PC and the details have been included as a supplemental table (Additional file 1: Table S1). Positive SL/PC was associated with linitis plastica (50.0% vs. 18.2%, *p* = 0.017) as well. All cases of moderately differentiated tumors and cT1 stage demonstrated were found to have negative SL/PC. There were no differences in age, gender, or race between the +SL/PC and −SL/PC early stage groups (all *p* > 0.05).

**Table 2:** Patients with early stage GA (*n* = 56), +SL/PC vs −SL/PC

		SL/PC status		
		Both negative (*n* = 46)	SL and/or PC positive (*n* = 10)	*p* value
Histology	Well differentiated	0 (0.0%)	0 (0.0%)	0.098
	Moderately differentiated	12 (26.1%)	0 (0.0%)	
	Poorly differentiated	34 (73.9%)	10 (100.0%)	
	Undifferentiated/anaplastic	0 (0.0%)	0 (0.0%)	
Signet ring cells		25 (54.3%)	8 (80.0%)	0.172
Linitis plastica		8 (18.2%)	5 (50.0%)	0.017
EUS T stage	T1b	2 (4.3%)	0 (0.0%)	1.000
	T2	44 (95.7%)	10 (100.0%)	

**Fig. 2:**
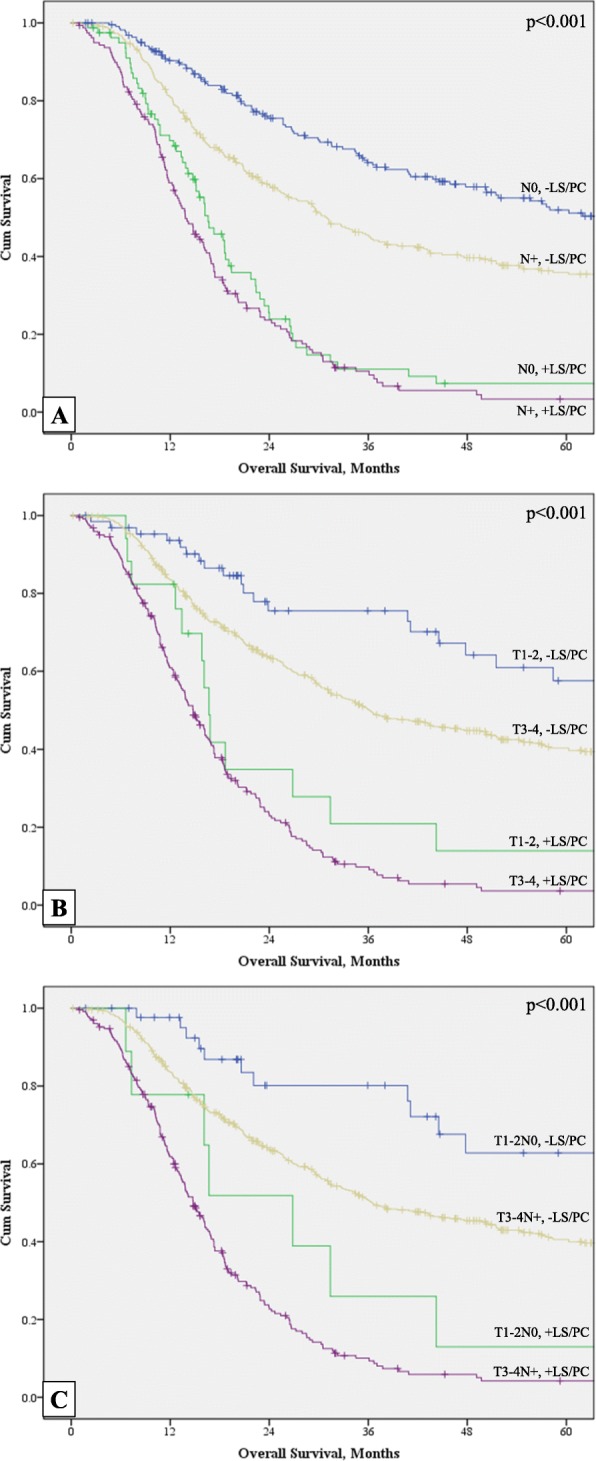
Kaplan-Meier survival analyses (*n* = 867). **a** Effect of SL/PC on survival stratified by N status. **b** Effect of SL/PC on survival stratified by T stage. **c** Effect of SL/PC on survival stratified by T and N status

The median OS for the entire cohort was 26.6 months (95% CI 23.7–29.4). Figure 2 depicts the Kaplan-Meier survival analyses as stratified by SL/PC status and clinical T and N stage. Notably, the presence of positive SL and/or PC significantly affects OS (*p* < 0.001) regardless of T or N stage. Those with clinically early T or N stage with positive SL/PC had significantly worse survival compared with those with advanced T or N stage disease but negative SL/PC. The median OS in early stage patients with +SL/PC was 26.8 months (95% CI 12.8–40.9), compared with 36.5 months (95% CI 28.3–44.8) in clinically late stage patients with negative SL/PC. The 5-year OS in early GA patients with +SL/PC was 13.0% compared with 62.8% in those with −SL/PC (*p* < 0.001). The 5-year OS in late GA patients was 40.6% in those with −SL/PC and 4.2% in those with +SL/PC.

## Discussion

The presence of peritoneal disease has a significant impact on prognosis in multiple gastrointestinal and gynecologic malignancies. In gastric cancer, peritoneal disease typically results in death after a short period of time, with a median interval of less than 1 year in most reports [7]. Despite the theory that peritoneal spread typically occurs in locally advanced tumors, our current study finds that peritoneal staging identifies metastatic disease in a significant number of patients with relatively early stage GA. The presence of peritoneal disease has a greater impact on survival than clinical T or N stage. Additionally, we found that all peritoneal spread in early stage cancers were in patients with poorly differentiated histologic grade.

Multiple theories of peritoneal spread from gastrointestinal cancers have been described in the literature [15, 28, 29]. The first step of the multi-step process is the establishment of free cancer cells within the peritoneal cavity and has been described by multiple methods. Possible mechanisms include spontaneous or iatrogenic perforation of the primary tumor, or spillage of tumor cells from transected lymphatic channels or blood vessels at the time of surgical resection [28]. Other theories include cancer cell spread via lymphatic or hematogenous routes [15, 29]. The most supported mechanism of tumor spread to the peritoneum, however, is by exfoliation of free cancer cells from a primary tumor which has invaded through the serosa [15]. Serosal invasion by the primary tumor has been shown to be associated with higher rates of peritoneal recurrence [30, 31]. By this logic, it seems that locally advanced tumors by clinical staging (cT3-T4, cN+) would likely gain the most benefit from peritoneal staging prior to definitive resection. Challenging this theory, our study demonstrated a positive SL/PC in 17.9% of early stage (cT1-T2, cN0) tumors.

The release of free cancer cells requires failure of adherence molecules, most notably E-cadherin, in order to detach from the serosal surface. It has been demonstrated previously that gastric cancers that are negative for E-cadherin expression are more likely to be of poorly differentiated histology and also have higher rates of lymph node metastasis, peritoneal recurrence, and poorer survival outcomes [32]. This is consistent with our findings that of the early cancers, all cases of +SL/PC were seen in poorly differentiated tumors. Bando et al. investigated rates of positive PC in patients at the time of surgical resection and similarly demonstrated higher rates of positive PC in undifferentiated tumors [11]. However, a large study, by Feng et al, showed no significant difference in survival based on differentiation status. While it is noteworthy that all patients with +SL/PC in the early stage group were of poor differentiation, our limited sample size and the lack of well-differentiated tumors in this group prevent us from drawing definitive conclusions about the relationship between differentiation status and positive peritoneal staging or survival. This may be an area of interest for future research.

As the presence of peritoneal disease confers such a poor prognosis, it is important to weigh the benefit, if any, with the potential morbidity of definitive resection in this patient population. Current NCCN guidelines recommend systemic chemotherapy or best supportive care for patients with gastric cancer metastasis to the peritoneum [14], as it is uncommon that the benefits are felt to outweigh the risks of a definitive resection in these patients. One study found that in 25% of patients with gastric cancer being considered for definitive resection, preoperative staging of the peritoneum with laparoscopy and cytology resulted in changes to the patient’s treatment plan to a more palliative approach [12]. In a prior study, patients who underwent surgery for curative intent were most often found to recur with disease isolated to the peritoneum (~ 50%) [5]. This likely indicates a missed initial detection of occult peritoneal disease and carries important implications as an initial laparoscopy and peritoneal cytological analysis may have prevented unnecessary surgery. There is also the potential for a patient with positive PC to revert to negative PC after neoadjuvant treatment, leading to consideration for definitive resection and the potential for long-term survival. Mezhir et al. studied the outcomes of gastric cancer patients with positive peritoneal cytology prior to preoperative chemotherapy [4]. Of the patients in the study who underwent repeat peritoneal staging, 56% converted to negative cytology and demonstrated significantly improved disease specific survival compared with those with persistently positive cytology. Most of these patients ultimately underwent potentially curative gastrectomy [4]. Lorenzen et al. similarly demonstrated a 37% conversion rate from positive PC to negative PC after neoadjuvant chemotherapy, with again an improved survival compared with those with persistently positive PC [33]. Finally, a study from our institution evaluating gastric cancer patients with positive PC found that a very small number of patients treated with neoadjuvant chemotherapy or chemoradiation can ultimately undergo curative resection, with an improvement in survival [7]. Additionally, there has been growing interest in the potential benefits of hyperthermic intraperitoneal perfusion with chemotherapy (HIPEC) as a treatment option for gastric cancer patients with metastasis limited to the peritoneum [34–36]. A phase II clinical trial evaluating HIPEC in patients with low-volume peritoneal carcinomatosis or positive PC demonstrated clearance of peritoneal disease in 37% of patients, with some going on to have definitive resection [36]. These studies underscore the importance of identifying patients with radiologically occult peritoneal disease in order to spare them the morbidity of a resection upfront and afford them the potential benefits of novel systemic and investigational therapies.

Currently, NCCN guidelines for gastric and gastroesophageal cancer recommend SL and PC in patients with clinical stage T1b or higher [14]. To our knowledge, the data supporting this practice in early stage disease is lacking; however, our current study confirms that SL and PC identifies peritoneal disease in a significant number of patients with early stage GA, particularly in those with poorly differentiated adenocarcinoma. Specifically, we did not identify any early clinical stage patients with well or moderately differentiated tumors to have positive SL/PC. Those with positive SL/PC also had high rates of signet ring histology (80%) and linitis plastica (50%) compared with those with negative SL/PC. Early stage patients with these high-risk features may represent a subgroup of patients in whom independent SL/PC would be most beneficial. The number of patients in our study without these high-risk features was not large enough to make a conclusive recommendation against SL/PC staging in this population.

Another aspect our study reveals is the importance in recognizing the established limitations of preoperative staging in reliably identifying early stage disease. Ikoma et al. demonstrated limited accuracy of EUS at determining actual T stage, as well as both computed tomography (CT) and EUS having low sensitivities in determining N status [37]. We feel that under-staging may have contributed to the relatively large number of early stage patients with peritoneal carcinomatosis, and therefore we have limited confidence in foregoing peritoneal staging based on preoperative staging.

There are limitations to our study. First, it was performed at a tertiary care center, where the typical patient population has advanced cancer. This is evidenced by the relatively small number of early stage gastric cancer patients that were included in the study. This bias may limit our study’s generalizability to all cancer centers treating gastric cancer. Additionally, the retrospective design of the study introduces its own potential selection biases, preventing control of multiple variables which could potentially influence the patient outcomes. For example, we did not examine all patients with cT1/N0 disease, only those that had SL and PC thus possibly creating selection bias, including primarily patients with high-risk features and thus increased clinical suspicion for peritoneal metastasis. However, all patients with clinical T2 lesions undergo SL/PC at our institution. There were only two patients with T1b lesions that underwent SL/PC in our study. Both of these patients had other high-risk features. Although not part of our institutional protocol, SL/PC consideration is recommended by current NCCN guidelines for anyone with a T1b lesion. Ultimately, the decision for preoperative SL/PC is at the discretion of the treating physicians. Practices regarding SL have evolved over the years, but it has remained an important aspect of the management of gastric cancer at our institution. Despite the possibility of evolving practices over the years, this study only included patients who underwent SL and PC. Despite these limitations, we demonstrate the significant negative impact that the presence of peritoneal disease has on survival outcomes in gastric cancer patients, regardless of clinical T or N stage.

## Conclusion

Peritoneal staging identifies metastatic disease in a significant number of patients with early stage GA. The presence of peritoneal disease has a greater impact on survival than clinical T or N stage. Given its poor prognosis and alternate therapy options, independent SL and PC should be considered in patients with GA and early clinical stage disease.

## Supplementary information

**Additional file 1:Table S1.** Radiological features of the preoperative imaging in ten early clinical stage patients with +SL/PC.

## Data Availability

The dataset used and analyzed during the current study are not publicly available due to patient confidentiality, but are available from the corresponding author on reasonable request.

## Abbreviations

EUS: Endoscopic ultrasonography/endoscopic ultrasound
GA: Gastric adenocarcinoma
H&E: Hematoxylin and eosin
HIPEC: Hyperthermic intraperitoneal perfusion with chemotherapy
IHC: Immunohistochemistry
NCCN: National Comprehensive Cancer Network
OS: Overall survival
PC: Peritoneal cytology
SL: Staging laparoscopy
