# Supplementary material for: Staging laparoscopy and peritoneal cytology in patients with early stage gastric adenocarcinoma
Source: World J Surg Oncol. 2020 Feb 17;18:39. doi: 10.1186/s12957-020-01813-y (PMC7026970; doi:10.1186/s12957-020-01813-y)
Supplement: Supplementary file 1 — Additional file 1:Table S1. Radiological features of the preoperative imaging in ten early clinical stage patients with +SL/PC. [file 12957_2020_1813_MOESM1_ESM.docx]

| Patient | Preoperative Staging CT and/or PET scan impression |
| --- | --- |
| 1 | Gastric wall thickening; no definite evidence of metastatic disease |
| 2 | Mass-like mural thickening in distal stomach; no evidence of regional or distant metastatic disease |
| 3 | Fairly diffuse gastric wall thickening; no definite evidence of metastatic disease |
| 4 | Diffuse thickening of gastric body; non-specific enhancing lymph node within gastrohepatic ligament |
| 5 | Gastric wall thickening; mild perigastric adenopathy; indeterminate retroperitoneal lymph nodes. |
| 6 | Irregular hypermetabolic thickening of the stomach wall; no hypermetabolic metastatic disease noted. |
| 7 | Thickening in the gastric wall; no evidence of significant adenopathy, no liver or splenic metastasis. |
| 8 | Ulceration in gastric body is not identified on this study; no evidence of enlarged left gastric lymph nodes or perigastric lymph nodes; no evidence of metastatic disease. |
| 9 | Diffuse thickening of the stomach; no evidence of retroperitoneal adenopathy. |
| 10 | Gastric wall is thickened; no definite peritoneal implant is seen; small retroperitoneal nodes, but no definite adenopathy; there is no hepatic metastasis. |
